# The Role of Mathematical Models in Immuno-Oncology: Challenges and Future Perspectives

**DOI:** 10.3390/pharmaceutics13071016

**Published:** 2021-07-02

**Authors:** Aymara Sancho-Araiz, Victor Mangas-Sanjuan, Iñaki F. Trocóniz

**Affiliations:** 1 Department of Pharmaceutical Technology and Chemistry, School of Pharmacy and Nutrition, University of Navarra, 31009 Pamplona, Spain; aaraizsanch@alumni.unav.es (A.S.-A.); itroconiz@unav.es (I.F.T.); 2 Navarra Institute for Health Research (IdiSNA), 31009 Pamplona, Spain; 3 Department of Pharmacy and Pharmaceutical Technology and Parasitology, University of Valencia, 46100 Valencia, Spain; 4 Interuniversity Research Institute for Molecular Recognition and Technological Development, 46100 Valencia, Spain

**Keywords:** immuno-oncology, PK/PD, mathematical modeling, bottom-up approach, middle-out approach, top-down approach

## Abstract

Immuno-oncology (IO) focuses on the ability of the immune system to detect and eliminate cancer cells. Since the approval of the first immune checkpoint inhibitor, immunotherapies have become a major player in oncology treatment and, in 2021, represented the highest number of approved drugs in the field. In spite of this, there is still a fraction of patients that do not respond to these therapies and develop resistance mechanisms. In this sense, mathematical models offer an opportunity to identify predictive biomarkers, optimal dosing schedules and rational combinations to maximize clinical response. This work aims to outline the main therapeutic targets in IO and to provide a description of the different mathematical approaches (top-down, middle-out, and bottom-up) integrating the cancer immunity cycle with immunotherapeutic agents in clinical scenarios. Among the different strategies, middle-out models, which combine both theoretical and evidence-based description of tumor growth and immunological cell-type dynamics, represent an optimal framework to evaluate new IO strategies.

## 1. Introduction

Cancer is one of the leading causes of death worldwide with a growing incidence due, in part, to increased life expectancy and diagnosis. Research advances in molecular biology have led to an expansion in the knowledge about the etiology of cancer, means of increasing the number of targets, as well as the therapeutic strategies available. Immuno-oncology (IO) focuses on stimulating the patient’s own immune system to act selectively against tumor cells treatments through the production of sustainable T cell responses and, thereby, diminishing the toxicity linked with traditional treatments [1,2,3]. In this sense, IO has revolutionized the cancer therapeutic paradigm, especially in non-solid hematological tumors and metastatic cancer, with an exponential growth in the number of scientific publications since 2016 and becoming, in 2021, the therapeutic oncology strategy with the highest number of approved drugs by the Food and Drug Administration (FDA) and the European Medicines Agency (EMA) (Table 1).

The tumor microenvironment (TME) comprises a heterogeneous population of cancer cells, as well as a variety of resident and infiltrating host cells, secreted factors and extracellular matrix proteins. The study of TME has provided insight on the possible factors controlling tumor progression and determining if the primary tumor eradicates, metastasizes or establishes dormant micrometastases [2]. Factors such as transforming growth factor-β (TGF-β), interleukin (IL)-4, programmed cell death 1 (PD-1), and programmed death ligand 1 (PD-L1) have been identified as fundamental elements developed by the tumor itself to escape the immune response [3].

Despite the successful path undertaken, the complexity associated with pharmacological agents, the pathophysiology of the tumor and the immune response are factors that may explain the absence of clinical response or the appearance of resistance mechanisms in at least 60% of patients [6]. Given the complexity of the tumor-immunogenicity tandem, pharmacometrics is being considered as a potential tool to bring together and understand the interplay of multiple factors affecting the pathophysiological and drug response, together with the identification of the different sources of variability. Traditional population pharmacokinetic and pharmacodynamic (PK/PD) models, which in essence are fully data-driven, typically connect modulation of pharmacological targets to clinical outcomes through empirical models, but do not fully capture all the existing mechanistic descriptions. In contrast, quantitative system pharmacology (QSP) follows the bottom-up paradigm and integrates knowledge of molecular and cellular interactions involved in the tumor growth and immune response. Regardless of the modelling approach, the use of model-based strategies has become an essential tool in the decision-making process to efficiently guide the selection of therapeutic agents, dosing regimens, biomarkers and/or clinical outcomes during the drug discovery and development process [7,8,9].

Several articles have already reviewed the different QSP models developed in the IO area and their role in drug development [10,11,12,13,14]. Those types of models are hard to apply to in vivo data with the aim of estimating individual parameters and correlating them with patients’ specific characteristics. The current review is intended to highlight modelling efforts that stretch the granularity of the in vivo longitudinal data, and can serve as a template for semi-mechanistic PKPD modelling in clinical trials. Prior to discussing modelling cases, we provide a comprehensive summary of different pharmacological targets for a better understanding of the models’ structures.

## 2. Current and Emerging Targets in Immuno-Oncology

Immune checkpoint inhibitors (ICIs) currently represent the most promising cancer therapeutics, producing durable responses in 40–50% of the patients administered them as monotherapies [15,16,17,18,19,20]. Among the different checkpoints expressed by cancer cells, cytotoxic T-lymphocyte-associated protein 4 (CTLA-4) and PD-1 are the most explored checkpoints for ICI-based therapeutics. Nevertheless, single drug checkpoint inhibitors did not achieve adequate response rates or prolonged disease control for ovarian [21,22,23], prostate and pancreatic cancers [22,24,25]. In this sense, current efforts are focused on developing predictors of response to immunotherapy and rational therapeutic combinations of current immune checkpoint inhibitors (PD-1 and CTLA-4) with novel checkpoints, cellular immunotherapies and delivery strategies, to improve the success rates in oncology [15].

### 2.1. Current Immune Checkpoint Inhibitors

**Figure 1:**
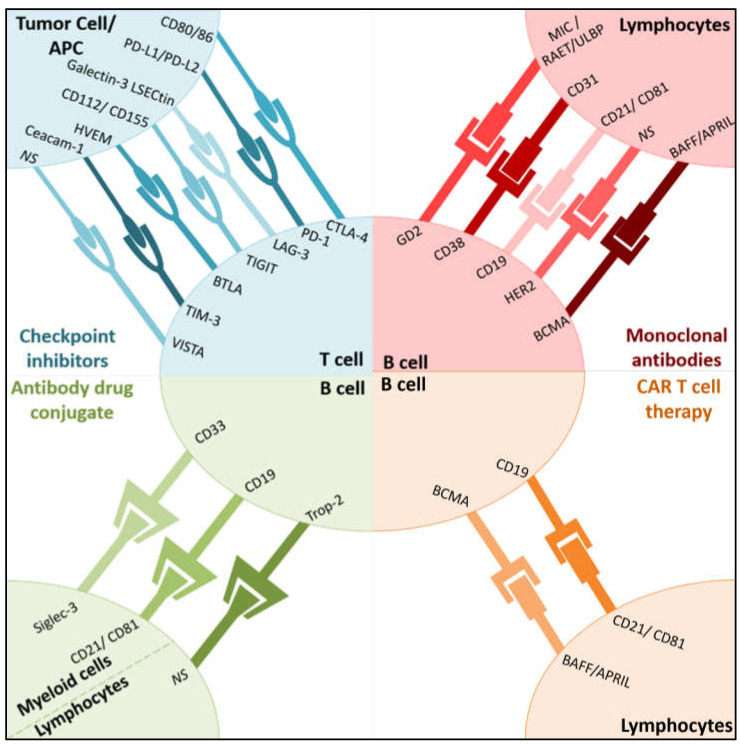
Main target and receptor of immunotherapies. Targets (inner circle) and receptors (outer circles) of the immune checkpoint inhibitors, monoclonal antibodies, antibody drug conjugates and CAR T-cell therapies described in Table 2 and the main text are summarized in the figure. Abbreviations: Siglec-3—sialic acid binding Ig-like lectin 3, BCMA—B-cell maturation antigen, MIC—MHC class I-related chain, RAET—retinoic acid early transcript, ULBP—UL16 binding protein, BAFF—B cell activation factor, APRIL—a proliferation-inducing ligand, NS—not specified; other abbreviations are found in the text.

CTLA-4 is a checkpoint of the immune system involved in the negative regulation of T cells at early immune response and is upregulated in activated T cells and expressed on regulatory T cells (Figure 1). The interaction between CTLA-4 and B7 molecules leads to an inhibitory signal to T cells and prevents the co-stimulatory signal transduction [26]. Anti-CTLA-4 agents are able to decrease regulatory T cells (Tregs) in the TME [27,28] and promote the activation of effector cells by blocking the inhibitory axis. Following CTLA-4, the PD-1/PD-L1/PD-L2 axis was the next prime target that received more attention for immune checkpoint therapies. These receptors are expressed on the cell surface of immune cells, dendritic cells and cancer cells. Similarly to CTLA-4, PD-1 ligation inhibits signaling downstream of the T cell receptor [29] (TCR) (Figure 1). Therefore, the development of antagonist against PD-1 or PD-L1 has emerged as a valuable therapeutic strategy to enhance the activity of T cells against tumoral cells, especially for melanoma, non-small cell lung cancer, renal cell carcinoma or Hodgkin lymphoma, among others, where durable responses were observed in 20–40% of patients [30,31].

### 2.2. Novel Immune Checkpoint Inhibitors

Tim-3 (T cell immunoglobulin and mucin domain 3) is highly expressed on dysfunctional or “exhausted” T cells in chronic viral infections and cancer (Figure 1). Tim-3 can interact with its different ligands, resulting in the inhibition of innate immune responses to nucleic acids, the induction of Th1 cells’ apoptosis and T cell tolerance, and the promotion of cross-presentation by dendritic cells (DCs) [32]. It has been shown that Tim-3 blockade increases cytokine production and tumor-antigen specific T cells’ proliferation [33,34,35].

T cell immunoglobulin and ITIM domain (TIGIT) is present in cytotoxic CD8+ T cells, regulatory T cells and other immune cells [36,37] (Figure 1). The dual blockade of TIGIT and PD-1 has proven to enhance CD8+ T cell expansion and cytotoxic activity against tumor cells [37]. Furthermore, lymphoid cells (cytotoxic and regulatory T cells) express the co-inhibitory receptor LAG3 (Figure 1), which promotes the inactivation of regulatory T cells. This process inhibits the T cell killing activity over tumor cells [38,39,40]. Therefore, the blockade of LAG-3 helps to restore the immune activity of T cells and, consequently, their anti-tumor activity. Several clinical trials have shown the potential role of LAG-3 inhibitors as an adjuvant therapy for melanoma, prostate and metastatic breast cancer, with tumor response rates of 50% [41,42,43].

V-domain Ig suppressor of T cell activation (VISTA) is a transmembrane protein involved in antitumor immunity through the negative regulation of T cells [44] (Figure 1). VISTA is an immune checkpoint gene that is structurally similar to PD-L1 and PD-L2 [45], and its over-expression in tumor cells inhibits T cell proliferation and cytokine production, resulting in tumor evasion [46,47,48]. High levels of VISTA have been identified in several malignant tumors, including oral squamous cell carcinoma, gastric carcinoma, hepatocellular carcinoma, prostate carcinoma, and melanoma [49,50].

B and T lymphocyte attenuator (BTLA) is a recently investigated inhibitory receptor, present in lymphoid cells, with promising preclinical results [51] (Figure 1). BTLA has structural and functional similarities with CTLA-4 and PD-1, and it has been found to be highly expressed in tumor antigen-specific CD8+ T cells of melanoma patients after peptide vaccinations. Preclinical evaluations in melanoma evidence the promising activity of monoclonal anti-BTLA antibodies by leading the promotion of T cell immune response [52,53].

### 2.3. Adoptive Cellular Immunotherapy

Adoptive cellular immunotherapy is an innovative and recently developed treatment strategy of promising success in the treatment of cancer patients [54], which aims to stimulate durable anti-tumor immune activity. These strategies include tumor-infiltrating lymphocytes (TIL), gene modified T cells expressing novel T cell receptors (TCR) and chimeric antigen receptors (CAR) [55,56].

CAR T cell therapies on the cell surface transmembrane protein of B cells’ CD19, the most studied target [57], have emerged as a promising tool in the management of hematologic malignancies [57,58] (acute lymphoblastic leukemia, diffuse large B cell lymphoma, chronic lymphocytic leukemia, and B cell non-Hodgkin lymphomas) (Figure 1). Despite the efficacy rates (50–90%) [59] of CAR T cell therapies on hematological malignancies observed in clinical trials [59,60,61], safety concerns (neurotoxicity, hepatotoxicity and multi-organ failure [58,62,63,64]) and tumor relapses have been identified [65,66]. In this sense, antigen escape resistance mechanisms have been recognized as a relevant factor to explain tumor relapse. This mechanism is enhanced in solid tumors, which might explain the reduced efficacy observed.

The use of TIL therapy has also emerged as an alternative tool to increase response rates and/or to reduce relapse rates, especially in metastatic melanoma and testis cancer patients [67,68,69,70,71,72,73,74]. Autologous lymphocytes are removed from the patient, externally expanded and re-infused to the patients to promote the immune response against tumor cells. Investigations are currently ongoing to evaluate the efficacy and safety of TIL administered as a monotherapy or in combination for different cancer treatments [75,76,77].

## 3. Mathematical Approaches Integrating Cancer Immunity Cycle with Immuno-Oncology Therapies

**Figure 2:**
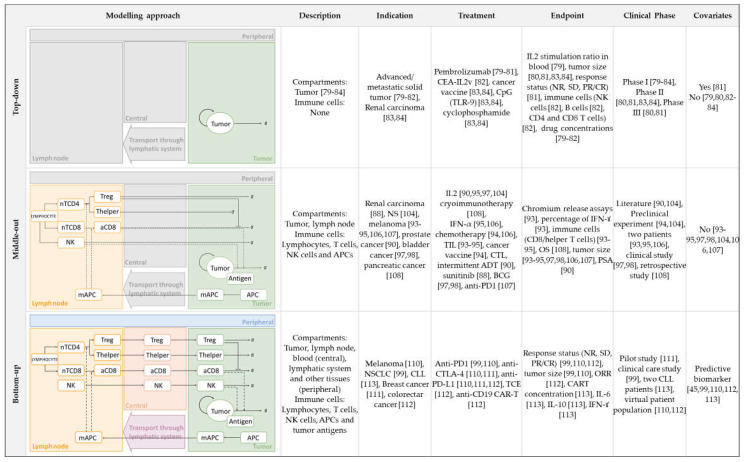
Characteristics of the modelling approaches developed in immuno-oncology. Dashed black arrows indicate activation, solid blocked arrows indicate inhibition, solid sharped arrows indicate transit between compartments, and ∅ indicates death. Colored compartments are used to depict modeling approaches that are contrary to those in the grey compartments. Abbreviations: IL—Interleukin, NR—non-responders, SD—stable disease, PR—partial response, CR—complete response, NK—natural killer, TIL—Tumor infiltrate lymphocyte, CTL—cytotoxic T lymphocytes, ADT—androgen deprivation therapy, *CIK*—cytokine-induced killer cell, BCG—Bacillus Calmette Guérin, ORR—overall response rate, OS—overall survival, PSA—prostate specific atnigen, NSCLC—non-small cell lung cancer, CLL—chronic lymphocytic leucemia, TCE—T-cell-engager, CAR—chimeric antigen receptor.

Mathematical modelling has broadly been used in support of preclinical and clinical research, as well as in decision-making in the oncology field [12]. This review will focus on those mathematical models that use ordinary differential equations to describe the IO system dynamics and that have been applied to clinical data. Additionally, we will divide the different works according to the modelling approach used: (i) top-down data-driven models built predominantly on the observed clinical data, and with a reduced number of parameters and equations leading to an empirical description of the biological system; (ii) bottom-up models based on knowledge about the human body and that are, therefore, as mechanistic as possible, utilizing in vitro as well as preclinical and clinical information as input data; and (iii) models that utilize a middle-out approach, combining bottom-up (model) and top-down (data) systems and applying different modeling strategies (Figure 2).

### 3.1. Top-Down Modelling and Simulation Approaches

Pharmacokinetic/pharmacodynamic (PK/PD) modeling has been used in the development of IO agents such as immune checkpoint inhibitors [78], monoclonal antibodies, toll-like receptor (TLR) agonists or cancer vaccines. As an example, PK/PD models have played an important role in supporting the dose setting and characterization of pembrolizumabs’ clinical pharmacology, a monoclonal antibody directed to PD-1 receptors. In particular, Elassaiss-Schaap et al. [79] developed a PK and a PK/PD model describing the pembrolizumab concentrations and doses at which maximal target engagement was achieved (Figure 2). As a major assumption, the IL-2 stimulation ratio in blood was considered as a surrogate for target engagement in the tumor, and, thus, a potential marker for antitumor efficacy. Similarly, Lindauer et al. [80] built a translational PKPD model that aimed to optimize dose setting in early clinical development in oncology, integrating in vitro, preclinical and clinical (Phase I, Phase II and Phase III clinical trial) data (Figure 2). The components of the final structure are: an empirical model for pembrolizumab PK in plasma, a mechanistic tissue compartment representing the site of drug action, a mechanistic binding model for drug–receptor interaction, and a tumor growth model. Finally, Chatterjee et at. [81] developed two top-down models describing the tumor size changes in melanoma patients (Phase I, Phase II and Phase III). Both models show the lack of clinically relevant impact of pembrolizumab exposure on response rate. Contrary, baseline disease, BRAF mutation, the degree of PD-1 receptor and ipilimumab treatment history were identified as possible predictors of individual variability. One of the limitations of this analysis for clinical extrapolation of the simulations is that the model does not consider dropouts (patients that discontinued the clinical trial), which could potentially impact the results. In spite of the different modeling approaches, equations and assumptions, all these models were developed for the same therapeutic agent and the same study population, and, thus, we can also find some similarities. In particular, these works described tumor growth following a simple exponential model (dTSdt=a TS) with a constant growth rate (a) ranging from 0.0017 to 0.0088 1/day and an initial tumor size ($TS_{0}$) estimated to be between 23.4 and 41.5 mm^3^ (Table 2).

A different PKPD model was developed by Ribba et al. [82] to guide a dose escalation study design of cergutuzumab amunaleukin (CEA-IL2v), a monoclonal antibody directed against carcinoembryonic antigens. In this study, a relation between drug plasma concentration (PK) and immune cell count (PD), including NK cells, CD4, CD8 T cells, was established using data from 74 patients with advanced and/or metastatic solid CEA+ tumors. Therefore, limited validation with clinical data was performed during model development. Besides, as the model equations describe drug, target (immune cells expressing IL2 receptor in blood) and complex drug–receptor concentrations, instead of tumor growth dynamics, the schematic representation does not fit the structure of Figure 2.

The mathematical models described until this point are focused on dealing with a specific pharmacological question such as the lowest effective dose to be used in a clinical trial [80], the optimal dosing schedule [82] or the quantification of the exposure–response relationships determining the efficacy of a certain therapeutic agent [81]. However, they do not consider the interactions between the immunological system and cancer cells. In this sense, more mechanistic models that aim to incorporate more components of the biological system can help to understand the mechanisms of actions of immunotherapies (Figure 2).

Parra-Guillen et al. [83,84] developed a model combining mechanistic features, specifically tumor resistance mechanisms, and mixed effects to describe tumor growth dynamics after the administration of different combinations of an antitumor vaccine, a TLR-9 agonist (CpG), chemotherapy (cyclophosphamide) and IL-2. This approach is also an example of how the kinetics of the therapeutic agents can be analyzed and simulated in the absence of PK information. Plasma concentration–time profiles of a drug are usually necessary to establish a relationship between the administered dose and the kinetics of drug action [85]. However, is not always possible to collect all the required PK data and several models have been proposed. Even so, despite the fact that this model is based on preclinical data only, it was successfully applied to reproduce clinical outcomes from three different studies (Phase I and Phase II data) (Figure 2). Still, the simplistic description of the tumor and immune system interactions is a handicap to be generalized to other mechanisms of actions.

**Table 2:** Summary of system- and drug-related pharmacodynamic parameters estimated with clinical data and published in the literature to account for immuno-oncology treatments.

Parameter	Value	Units	Estimation	Indication	Treatment	**Ref.**
**Tumor**						
Tumor growth						
Lineal: *a*	$TS_{0}=1.16\times 10^{-6}\;b=0.354$	mmmm/day	Colon cancer	Colon cancer	IL-2	[84]
Exponential: a TS	$TS_{0}=41.5\;b=0.0088-0.0017$	$\mathrm{mL}\;{\mathrm{day}}^{-1}$	Melanoma patients [80]	Melanoma	Anti-PD1	[80]
	$TS_{0}\;shallow\;tumor=57.9\;TS_{0}\;deep\;tumor=23.4\;b=0.00267$	$\mathrm{mm}\;\mathrm{mm}\;{\mathrm{day}}^{-1}$	Melanoma patients [81]	Melanoma	Anti-PD1	[81]
Logistic: a TS(1−bTS)	$a=2.065\;\times \;10^{-1}\;b=2.145\times 10^{-10}$	${\mathrm{day}}^{-1}\;{\mathrm{cell}}^{-1}$	Renal carcinoma [86,87]	Renal carcinoma	Sunitinib	[88]
	AD: b=2.025 AI: a=0.006	$\mathrm{ng}/\mathrm{mL}\;{\mathrm{day}}^{-1}$	Prostatic cancer [89]	Prostate	Intermittent ADT + DC vaccine	[90]
Tumor cell kill by CD8						
$\mathrm{Fractional}:\;\frac{d{\left(CD8/TS\right)}^{l}}{s+{\left(CD8/TS\right)}^{l}}TS$	d=5.80l=1.36s=0.512−0.839	${\mathrm{day}}^{-1}$ None None	3 × 10^5^ B16-BL6 cells [91]/Human [92]	Metastatic melanoma	Chemotherapy + TIL	[93]
	d=1.88−2.34l=1.81−2.09s=0.25	${\mathrm{day}}^{-1}$ None None	3 × 10^5^ B16-BL6 cells [91]/Human [92]	Metastatic melanoma	Chemotherapy + TIL + IL2 + cancer vaccine	[94,95]
Linear: d CD8 TS	$d=1.1\times 10^{-6}$	${\mathrm{cell}}^{-1}\times {\;\mathrm{day}}^{-1}$	High grade gliomas patients [96]	Bladder	IL2 + BCG	[97,98]
Equation in [99]	$K_{death}=4$	${\mathrm{day}}^{-1}$	Assumed [99]	NSCLC	Anti-PD1	[99]
Tumor cell kill by NK cells						
c N TS	$c=\left(3.23\times 10^{-7}\right)$	${\mathrm{cell}}^{-1}\times {\mathrm{day}}^{-1}$	3 × 10^5^ B16-BL6 cells [91]/Human [92]	Metastatic melanoma	Chemotherapy + TIL	[93]
	$c=\left(2.9077\times 10^{-13}\right)$	${\mathrm{cell}}^{-1}\times {\;\mathrm{day}}^{-1}$	3 × 10^5^ B16-BL6 cells [91]/Human [92]	Metastatic melanoma	Chemotherapy + TIL + IL2	[100]
	$c=\left(6.41\times 10^{-11}\right)$	${\mathrm{cell}}^{-1}\times {\;\mathrm{day}}^{-1}$	3 × 10^5^ B16-BL6 cells [91]/Human [92]	Metastatic melanoma	Chemotherapy + TIL + IL2 + IFNα	[95]
CD8 cells						
Number of CD8 per microliter of blood						
	1000	-	CD4+ count of 640-1175/µL humans	Melanoma	Pembrolizumab	[80]
CD8 recruitment by tumor						
$\mathrm{By}\;\mathrm{CD}8\;\mathrm{killing}:\;\frac{Vmax\;TS^{2}}{K_{M}+TS^{2}}CD8$	$Vmax=\left(3.75\times 10^{-2}\right)\;K_{M}=2.0\times 10^{7}$	${\mathrm{day}}^{-1}\;{\mathrm{cell}}^{2}$	BCL 1 lymphoma of chimeric mice [91,92,93,94,95,96,97,98,99,100,101]/Human [92]	Metastatic melanoma	Chemotherapy + TIL	[93]
	$Vmax=\left(32.49\times 10^{-2}\right)\;K_{M}=\left(23.66-5.66\right)\times 10^{7}$	${\mathrm{day}}^{-1}\;{\mathrm{cell}}^{2}$	BCL 1 lymphoma of chimeric mice [91,101]/Human [92]	Metastatic melanoma	Chemotherapy + TIL + IL2 + IFNα	[95]
$\frac{Vmax\;TS}{K_{M}+TS}CD8$	$Vmax=\left(1.245\times 10^{-2}\right)\;K_{M}=2.019\times 10^{7}$	${\mathrm{day}}^{-1}\;{\mathrm{cell}}^{2}$	BCL 1 lymphoma of chimeric mice [91,92,93,94,95,96,97,98,99,100,101]/Human [92]	Metastatic melanoma	Chemotherapy + TIL + IL2	[100]
$\mathrm{By}\;\mathrm{BCG}\;\mathrm{killing}:\;\mathrm{By}\;\mathrm{CD}8\;\mathrm{killing}:\;\frac{Vmax\;Ag}{K_{M}+Ag}IL2$	$Vmax=1.45\times 10^{8}\;K_{M}=10^{10}\;Vmax=1.514\times 10^{6}\;K_{M}=10^{10}$	${\mathrm{cell}}^{-1}\times {\mathrm{day}}^{-1}\times \mathrm{IL}2^{-1}\;{\mathrm{cell}}^{-1}\;{\mathrm{cell}}^{-1}\times {\mathrm{day}}^{-1}\times \mathrm{IL}2^{-1}\;{\mathrm{cell}}^{-1}$	In vitro/Estimated bladder cancer patients [102] In vitro/Estimated bladder cancer patients [103]	Bladder	IL2 + BCG	[97]
CD8 activation by APCs						
$\frac{e\;APC}{g+APC}$	$e=20\times 10^{6}\;g=400\times 10^{6}$	$\mathrm{cells}\times {\mathrm{day}}^{-1}\;\mathrm{cells}$	Preclinical experiments [104] Prostate cancer [105]	Prostate	Intermittent ADT + DC vaccine	[90]
**NK cells**						
Production rate NK						
From circulating lymphocytes:e L	$e=8.68\times 10^{-10}$	$L\times {\mathrm{cell}}^{-1}\times {\;\mathrm{day}}^{-1}$	Preclinical experiments renal carcinoma [86,87]	Renal carcinoma	Sunitinib	[88]
NK recruitment						
$\mathrm{By}\;\mathrm{tumor}:\;\frac{Vmax\;TS^{2}}{K_{M}+TS^{2}}N$	$Vmax=\left(2.5\times 10^{-2}\right)\;K_{M}=2.02\times 10^{7}$	${\mathrm{day}}^{-1}\;{\mathrm{cell}}^{2}$	BCL 1 lymphoma of chimeric mice [91,92,93,94,95,96,97,98,99,100,101]/Human [92]	Metastatic melanoma	Chemotherapy + TIL + IFNα	[93,94,95]

TS—Tumor size, TS_0_—Initial tumor size, AD—androgen dependent, AI—androgen independent, IL—Interleukin, NK—natural killer, TIL—Tumor infiltrate lymphocyte, ADT—androgen deprivation therapy, CIK—cytokine-induced killer cell, BCG—Bacillus Calmette Guérin, NSCLC—non-small cell lung cancer, CLL—chronic lymphocytic leucemia, APC—antigen presenting cells, DC—dendritic cells, BCL—B cell leukemia.

### 3.2. Middle-Out Modelling and Simulation Approaches

Middle-out approaches aim to incorporate the main biological and pharmacodynamic mechanisms of the system while maintaining a simplified model structure. However, keeping the principle of parsimony represents a challenge since it is probable that some model parameters will be hard to estimate precisely. As a consequence, the integration of different pharmacometric techniques, such as the use of Bayesian priors based on previous knowledge to inform poorly estimated parameters, is warranted [106].

This strategy provides a quantitative platform for model development in clinical scenarios (Figure 2). The first attempt to describe the interactions between the immune system and cancer cells [101] was a model of two ordinary differential equations (ODEs) describing the dynamics of CD8 T cells and their effect on tumor cell killing. On this basis, and with the increasing experimental data available, models were expanded to include different entities, including immune cells and cytokines, and mechanisms such as receptor expression dynamics.

At first, aiming to incorporate cytokines’ function into the models, Kirschner and Panetta [104] built a three-ODE system addressing the potential of IL-2 and its effects on tumor relapse. Similarly, in a more recent work carried out by Isaeva et al. [107], the effects of chemotherapy and immunotherapy (IL-2 and IFN-α) were studied using a middle-out approach. A limitation of this analysis is that in order to reflect the different clinical outcomes, patients were conditionally divided into three groups characterized by tumor antigen expression, the strength of the immune response and the reaction to vaccination (Figure 2 covariates).

On the other hand, with the goal of adding new entities, de Pillis et al. [93] developed a very simple model that included three cell populations: tumor cells, natural killer (NK) cells and CD8 T cells. This work supported the relevance of considering multiple cell types in the overall anti-tumor immune activity. In spite of the model’s simplicity, it is able to fit data from two metastatic melanoma patients treated with tumor-infiltrating lymphocytes (TIL) after chemotherapy. An essential feature of this model is the cell killing term defining the interaction of tumor cells with either NK or CD8 T cells. Although for NK cells, a linear product (Table 2; tumor cells killed by NK cells) was sufficient to reproduce the experimental data, for CD8 T cells, a rational form was needed. In Table 2 (fractional tumor cell kill by CD8), the parameter d gives the maximum lysis rate, the exponent l represents how the lysis rate depends on the effector/target ratio, and s is the parameter affecting the steepness of the curve. This new term and the parameter values have been used in previous works [94,95,100]. Although the model fits the empirical data, its structure is still very simple and does not incorporate self-regulatory terms or down-regulation of the activated immune response, among other mechanisms. In a different approach, Perlstein et al. [108] incorporated memory T cells and the senescence and exhaustion mechanisms of PD-1/PD-L1 checkpoint blockade immunotherapy in their model. One of their limitations is that the model was adjusted to fit the clinically measured dynamics of just one reference patient from a hospital cohort suffering from metastatic melanoma. Furthermore, Xuefang et al. [109] developed a mathematical prognosis model for pancreatic cancer patients including not only cancer cells and CD8 T cells, but also pancreatic stellate cells, other immune cells (NK cells and helper T cells) and cytokines (IL-2, IFN-α and TGF-β). They assumed that survival time is the time taken for the cancer cell density to reach a certain threshold (500 cells per µL).

Some of the common biological assumptions of the previously described models include: (i) cancer cells grow logistically in the absence of an immune response; (ii) both NK cells and CD8 T cells are capable of killing cancer cells; (iii) both NK cells and CD8 T cells are activated by cancer cells; (iv) both NK cells and CD8 cells eventually become inactivated after some number of interactions with tumor cells; (v) as part of the innate system, NK cells are always present, but CD8 T cells are only present when tumor is present.

The aforementioned models incorporate an increasing number of immune cells. However, they do not include immune-suppressive components, which have been demonstrated to play a critical role in tumor evasion mechanisms. Tumors escape the immune-mediated elimination by producing substances, such as TGF-β and IL-10, that stimulate the expansion of immunosuppressive cells, particularly regulatory T cells (Tregs), MDSCs, and M2 macrophages. With the aim of including these mechanisms, de Pillis et al. [88] expanded their previous model and incorporated Tregs as the main immunosuppressive component. This analysis studies the anti-angiogenic effect of sunitinib as well as its ability to directly inhibit the immunosuppressive environment by reducing the number of Tregs.

Whereas, until this point, the new entities incorporated into the models included immune players or cytokines, other authors have focused on including different tumor cell clones. Mahasa et al. [110] used a middle-out approach, with a model structure able to fit the representation of Figure 2, to study the immune surveillance of tumors including immune cells, different tumor cell populations (naïve and resistant), and the complexes formed among these. The model describes how tumor cell populations escape and acquire resistance after the interaction with the immune system mediated by NK and CD8 T cells. In a different work, Portz et al. [90] extended the model proposed by Kirschner and Panetta [104] and developed a system of six ODEs in which tumor mass was divided into androgen dependent and independent cells. This approach was driven by the fact that patients were treated with androgen deprivation therapy, which prevents growth and induces apoptosis only of androgen dependent cells. On a similar basis, Bunimovich-Mendrazitzky et al. [98] developed a model considering two different populations of tumor cells—infected and uninfected tumor cells. They studied Bacillus Calmette–Guerin (BCG) immunotherapy for superficial bladder cancer patients. This work was later expanded [97] by adding IL-2. In both situations, they modeled the encounter of effector cells and cancer cells with proportional rate constant (d CD8 TS). Despite the fact that more mechanisms are incorporated, the limited validation with clinical data together with the remaining simple description of the tumor and immune system interactions makes the extrapolation of this model to other immunotherapies challenging.

**Figure 3:**
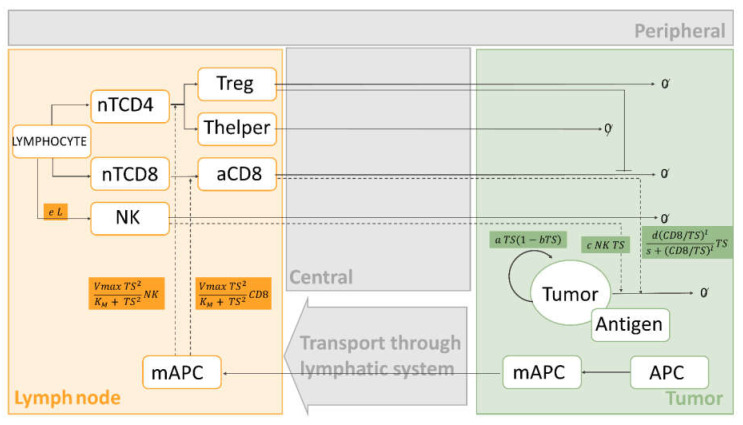
Middle-out approach model representation. The equations that are common to most of the middle-out approaches are described in the figure. Dashed black arrows indicate activation, solid blocked arrows indicate inhibition, solid sharped arrows indicate transit between compartments, and ∅ indicates death. Colored compartments are used to depict modeling approaches that are contrary those in the grey compartments. Abbreviations: *NK*—natural killer, nTCD4—naïve CD4 cells, nTCD8—naïve CD8 cells, Treg—regulatory T cells, Thelper—Helper T cells, *aCD8*—activated CD8 T cells, *mAPC*—mature antigen presenting cells, APC—immature antigen presenting cells, *TS*—tumor size, *L*—lymphocytes. Other parameters are presented in Table 2.

It can be noticed that the middle-out approaches include mechanistic features as part of relatively simple mathematical models. Furthermore, although most of the models follow a common structure, each of them focuses on those specific aspects that might be considered more relevant or with a bigger impact in immunotherapy. The mathematical framework depicted in Figure 3 summarizes the main mechanisms characterized in middle-out approaches, incorporating the mathematical functions proposed using clinical data. This framework integrates the current knowledge on several cell types and tissues with the main mathematical functions to provide a general scenario that could be used to develop future models based on available experimental evidence.

### 3.3. Bottom-Up Modelling and Simulation Approaches

Large-scale QSP platform models are able to integrate the current knowledge of the cancer immunity-cycle (Figure 2) by incorporating more mechanisms, different cell-types (tumor cells, innate and adaptive immune cells, and stromal cells) and diverse molecular components (cytokines, cell-surface receptors, etc.). However, these bottom-up modelling approaches are built up with a very large number of parameters and equations, and model calibration is usually challenging.

Popel’s group developed one of the first full-scale QSP platforms in IO, describing cancer cells, the dynamics and development of antigen presenting cells (APCs), T cells (naïve, primed, effector and regulatory) and myeloid derived suppressor cells (MDSCs) in the tumor, the tumor draining lymph node, the blood and other tissues. This framework has been validated for melanoma, breast cancer, and NSCLC patients treated with anti-PD1, anti-PD-L1, anti-CTLA4 and epigenetic inhibitors [99,111,112,113]. Moreover, the cellular interactions of TCR, MHC I, CD28, CD80, CD86, PD1, PD-L1, PD-L2, and CTLA4 surface receptor were considered. In these works, rather than developing a model from scratch in each particular situation, a platform model was created and then used to sequentially add components and/or adapt it to other tumor types. Wang et al. [47] studied the combination of entinostat (histone deacetylase inhibitor) with ICIs in breast cancer patients incorporating PD-L1 expression and tumor mutational burden (TMB). Then, Jafarnejad et al. [99] included a detailed model for APC antigen presentation, and Milberg et al. [111] proved that this platform was able to predict longitudinal tumor size profiles and the number of patients showing partial or complete response for anti-PD1 and anti-CTLA4 combinations in melanoma.

A different QSP model for anti-CD19 chimeric antigen receptor T cells (CAR T) in a patient with chronic lymphocytic leukemia (CLL) was developed by Hardiansyah et al. [114]. Since we are dealing with a non-solid tumor, the model-based platform developed by the authors does not fit the bottom-up structure shown in Figure 2. In this case, the authors have included the dynamics of B-cells, effector and memory CAR T cells, and inflammatory cytokines (interleukin-6, Interleukin-10, and interferon gamma) in peripheral blood and tissue. The proposed model is able to describe the observed CART kinetic and pro-inflammatory cytokine profiles in a clinical scenario.

In bottom-up approaches, although the majority of model parameters are literature-based (Table 2), a selected set of parameters are adjusted using clinical data. Nevertheless, one of their main limitations in drug development is building confidence intervals for a very large number of parameters. In spite of this, QSP models have been demonstrated to be a valuable tool for deepening our understanding on how the mechanism of action connects to the clinical outcomes and, therefore, may serve as important model-based platforms to guide the development of, and personalize, treatment therapy.

## 4. Conclusions

The number of approved immunotherapies has grown exponentially in the past two years, particularly immune checkpoint inhibitors, monoclonal antibodies, antibody drug conjugates and CAR T-cell therapies. Despite the improvement in response rates, there is still a high percentage of patients that do not respond to these treatments. For this reason, current efforts are focused on finding new therapeutic targets and different combination strategies. In this sense, mathematical strategies have proven to be an efficient tool to characterize, select and predict optimal therapeutic alternatives in the field of IO. However, it is still necessary to develop a quantitative framework that allows the evaluation of two or more agents administered in combination and to identify their possible interactions. Among the different mathematical models proposed to describe the tumor and immune cells’ interactions, in this review, we have focused on those that use ordinary differential equations and have been applied to clinical settings. Additionally, models have been classified into top-down, middle-out or bottom-up approaches, according to the modelling strategy applied.

On the one hand, data-driven top-down models have been demonstrated to be a successful tool in clinical trials, for example, to predict the minimum efficacious dose of ICIs. However, one of the limitations is that due to the simplicity of such models, some assumptions have to be considered, for example, the simplification of the drug delivery process or the extrapolation of parameters from animals to humans. Furthermore, since the interactions of the tumor and the immune system are not considered, they cannot be generalized to therapeutic agents with other mechanisms of action or their combinations. On the other hand, QSP models simulate various biological processes and interactions on different tissues and, thus, can help to overcome the challenges of understanding the immune response dynamics and the interplay of tumor infiltration processes and tumor cell growth. Nevertheless, the large number of parameters, and the relatively small amount of observed data usually available, makes the development of these models very complex.

In between these two approaches, middle-out strategies offer theoretical and evidence-based description, representing an optimal framework for the evaluation of new strategies in IO. These models are based on experimental and/or clinical data while constraining the model structure to the current knowledge of the system. Therefore, this modeling strategy needs either data from specific biomarkers that allow the identification of immune cells’ dynamics, or an experimental design in which immune-modulators acting on different steps of the cancer-immunity cycle are studied. Moreover, a relevant aspect to be incorporated into mathematical models is the development of biomarkers capable of predicting degrees of response in cancer patients.

In this regard, the design of studies that allow the collection of informative longitudinal data, together with the integration of pharmacogenetics, can contribute to establishing early response indicators. On the whole, this work provides a schematic representation (Figure 2), including the description of tumor growth and immunological cell-type dynamics, as well as a range of model equations and parameters, with the aim of establishing an optimal theoretical framework for middle-out approaches, which may help to evaluate new IO strategies.

## Figures and Tables

**Table 1:** Approved immuno-oncology treatments by the FDA and EMA in 2020 and 2021.

Therapy	Date		Active Principle	Commercial Name	Company	Indication		Agency
**Checkpoint Inhibitor**	2020	January	Pembrolizumab	Keytruda	MSD	Bacillus Calmette–Guerin (BCG)-unresponsive, high-risk, non-muscle invasive bladder cancer (NMIBC) with carcinoma in situ (CIS) with or without papillary tumors who are ineligible for or have elected not to undergo cystectomy		FDA
		March	Durvalumab	Imfinzi	AstraZeneca	First-line treatment of patients with extensive-stage small cell lung cancer (ES-SCLC)		FDA
			Nivolumab + Ipilimumab	Opdivo/ Yervoy	Bristol-Myers Squibb	Hepatocellular carcinoma (HCC) who have been previously treated with sorafenib		FDA
		May	Nivolumab	Opdivo	Bristol-Myers Squibb	Metastatic non-small cell lung cancer (NSCLC) with epidermal growth factor receptor (EGFR) exon 19 deletions or exon 21 (L858R) mutations		FDA
			Atezolizumab	Tecentriq	Genentech	Unresectable or metastatic hepatocellular carcinoma who have not received prior systemic therapy		FDA
			Nivolumab + Ipilimumab	Opdivo/ Yervoy	Bristol-Myers Squibb	First-line treatment for patients with metastatic or recurrent NSCLC, with no epidermal growth factor receptor (EGFR) or anaplastic lymphoma kinase (ALK) genomic tumor aberrations		FDA
			Atezolizumab	Tecentriq	Genentech	First-line treatment of adult patients with metastatic NSCLC whose tumors have high PD-L1 expression with no EGFR or ALK genomic tumor aberrations		FDA
			Nivolumab + Ipilimumab	Opdivo/ Yervoy	Bristol-Myers Squibb	First-line treatment for patients with metastatic NSCLC whose tumors express PD-L1(≥1%) with EGFR or ALK genomic tumor aberrations		FDA
		June	Avelumab	Bavencio	EMD Serono	Maintenance treatment of patients with locally advanced or metastatic urothelial carcinoma (UC) that has not progressed with first-line platinum-containing chemotherapy		FDA
			Pembrolizumab	Keytruda	MSD	First-line treatment of patients with unresectable or metastatic microsatellite instability-high (MSI-H) or mismatch repair deficient (dmmr) colorectal cancer		FDA
						Recurrent or metastatic cutaneous squamous cell carcinoma (cscc) that is not curable by surgery or radiation		FDA
			Pembrolizumab	Keytruda	MSD	Unresectable or metastatic tumor mutational burden-high (TMB H) [≥10 mutations/megabase (mut/Mb)] solid tumors		FDA
		July	Atezolizumab	Tecentriq	Genentech	BRAF V600 mutation-positive unresectable or metastatic melanoma		FDA
		September	Nivolumab	Opdivo	Bristol-Myers Squibb	Nd		EMA
			Ipilimumab	Yervoy	Bristol-Myers Squibb	Nd		EMA
			Atezolizumab	Tecentriq	Roche	Nd		EMA
		October	Pembrolizumab	Keytruda	MSD	Relapsed or refractory classical Hodgkin lymphoma (chl)		FDA
			Pembrolizumab	Keytruda	MSD	Pediatric patients with refractory chl, or chl that has relapsed after 2 or more lines of therapy		FDA
			Nivolumab + Ipilimumab	Opdivo/ Yervoy	Bristol-Myers Squibb	First-line treatment for adult patients with unresectable malignant pleural mesothelioma		FDA
		November	Pembrolizumab	Keytruda	MSD	Locally recurrent unresectable or metastatic triple-negative breast cancer (TNBC) whose tumors express PD-L1 (CPS ≥ 10)		FDA
	2021	January	Nivolumab + Cabozantinib	Opdivo/ Cabometyx	Bristol-Myers Squibb/Exelixis	First-line treatment for patients with advanced renal cell carcinoma		FDA
		February	Cemiplimab	Libtayo	Regeneron Pharmaceuticals	First-line treatment of patients with advanced NSCLC whose tumors have high PD-L1 expression		FDA
			Cemiplimab	Libtayo	Regeneron Pharmaceuticals	Locally advanced and metastatic basal cell carcinoma		FDA
			Dostarlimab	Jemperli	GSK	Treatment of certain types of recurrent or advanced endometrial cancer		EMA
			Nivolumab	Opdivo	Bristol-Myers Squibb	Nd		EMA
		March	Atezolizumab	Tecentriq	Roche	First-line treatment of adult patients with metastatic NSCLC whose tumours have a PD-L1 expression ≥ 50% tumour cells or ≥ 10% tumour-infiltrating immune cells and who do not have EGFR mutant or ALK-positive NSCLC		EMA
			Pembrolizumab	Keytruda	MSD	Metastatic or locally advanced esophageal or gastroesophageal carcinoma who are not candidates for surgical resection or definitive chemoradiation		FDA
			Dostarlimab	Jemperli	GSK	Mismatch repair deficient recurrent or advanced endometrial cancer		FDA
			Nivolumab	Opdivo	Bristol-Myers Squibb	Advanced or metastatic gastric cancer, gastroesophageal junction cancer, and esophageal adenocarcinoma		FDA
			Nivolumab	Opdivo	Bristol-Myers Squibb	Malignant pleural mesothelioma		EMA
			Ipilimumab	Yervoy	Bristol-Myers Squibb	Malignant pleural mesothelioma		EMA
**Monoclonal Antibody**	2020	March	Isatuximab-irfc	Sarclisa	Sanofi	Multiple myeloma who have received at least two prior therapies including lenalidomide and a proteasome inhibitor		FDA
			Isatuximab-irfc	Sarclisa	Sanofi	Multiple myeloma		EMA
		May	Daratumumab + hyaluronidase-fihj	Darzalex Faspro	Janssen Biotech	Newly diagnosed or relapsed/refractory multiple myeloma		FDA
		July	Tafasitamab-cxix	Monjuvi	MorphoSys US	Relapsed or refractory diffuse large B-cell lymphoma not otherwise specified, including DLBCL arising from low grade lymphoma, and who are not eligible for autologous stem cell transplant		FDA
		August	Belantamab mafodotin-blmf	Blenrep	GSK	Relapsed or refractory multiple myeloma who have received at least 4 prior therapies, including an anti-CD38 monoclonal antibody, a proteasome inhibitor, and an immunomodulatory agent		FDA
		November	Naxitamab	Danyelza	Y-mAbs Therapeutics	Pediatric patients one year of age and older and adult patients with relapsed or refractory high-risk neuroblastoma in the bone or bone marrow demonstrating a partial response, minor response, or stable disease to prior therapy		FDA
		December	Margetuximab-cmkb	Margenza	MacroGenics	Metastatic HER2-positive breast cancer who have received two or more prior anti-HER2 regimens, at least one of which was for metastatic disease		FDA
	2021	March	Isatuximab-irfc	Sarclisa	Sanofi	Relapsed or refractory multiple myeloma who have received one to three prior lines of therapy		FDA
**Antibody Drug Conjugate**	2020	April	Sacituzumab govitecan-hziy	Trodelvy	Immunomedics	Metastatic TNBC who received at least two prior therapies for metastatic disease		FDA
	2021	April	Loncastuximab tesirine-lpyl	Zynlonta	ADC Therapeutics	Relapsed or refractory large B-cell lymphoma after two or more lines of systemic therapy, including DLBCL not otherwise specified, DLBCL arising from low grade lymphoma, and high-grade B-cell lymphoma		FDA
			Sacituzumab govitecan	Trodelvy	Immunomedics	Advanced urothelial cancer		FDA
			Sacituzumab govitecan	Trodelvy	Immunomedics	Unresectable locally advanced or metastatic TNBC who have received two or more prior systemic therapies, at least one of them for metastatic disease		FDA
**CAR T-Cell Therapy**	2020	June	Gemtuzumab ozogamicin	Mylotarg	Wyeth	Newly-diagnosed CD33-positive acute myeloid leukemia (AML) to include pediatric patients 1 month and older		FDA
		July	Brexucabtagene autoleucel	Tecartus	Gilead	Relapsed or refractory mantle cell lymphoma		FDA
	2021	January	Daratumumab + Hyaluronidase	Darzalex Faspro	Janssen Biotech	Newly diagnosed light chain (AL) amyloidosis		FDA
		February	Lisocabtagene maraleucel	Breyanzi	Juno	Relapsed or refractory large B-cell lymphoma after two or more lines of systemic therapy		FDA
			Isatuximab	Sarclisa	Sanofi	Multiple myeloma who have received at least one prior therapy		EMA
		March	Idecabtagene vicleucel	Abecma	Bristol-Myers Squibb	Relapsed or refractory multiple myeloma after four or more prior lines of therapy, including an immunomodulatory agent, a proteasome inhibitor, and an anti-CD38 monoclonal antibody		FDA
			Axicabtagene ciloleucel	Yescarta	Kite Pharma	Relapsed or refractory follicular lymphoma (FL) after two or more lines of systemic therapy		FDA

ND—not described; FDA—Food and Drug Administration; EMA—European Medicines Agency; GSK—GlaxoSmithKline; MSD—Merck Sharp and Dohme. Source: EMA [4] and FDA [5] webpages.
